# Collaborative research: population-based data and validation are necessary

**DOI:** 10.1093/bjs/znac100

**Published:** 2022-04-14

**Authors:** Martin Björck

**Affiliations:** Department of Surgical Sciences, Uppsala University, Uppsala, Sweden; Institute of Clinical Medicine, University of Tartu, Tartu, Estonia

Li and Bhangu^1^ are pushing through an open door suggesting that multicentre, international research collaboration is wonderful, yet it challenges individual author accountability, and may devalue the hard yards completed by those who organized, funded, analysed, and reported the studies. Organizers struggle with the conflict between motivating colleagues to join and yet sticking to established authorship rules (ICMJE.com). Instead of classifying all contributors as authors, it may be more correct to diversify contribution into authors, collaborators, and acknowledgements. Authorship carries important responsibilities. The Swedish Research Council nominated me to investigate the research fraud committed by Paolo Machiarini and colleagues^2^. Many of the co-authors who had put their names to the six fraudulent papers regretted doing so. Fortunately, there were about 50, not 15 000.

Authorship rules are not the only elephant in the room. Selection bias is another one. Li and Bhangu^1^ argue that ‘This makes the findings representative of wider populations, meaning that the results are generalizable and relevant to more patients’ and ‘the most recent and prominent example being the COVID-19 pandemic’. I will outline my challenges to this opinion.

Big data and speed in scientific publishing are not necessarily beautiful. At the start of the pandemic, the editors of the *European Journal of Vascular and Endovascular Surgery* published an editorial^3^ underlining the need for research initiatives amidst and after the COVID-19 pandemic. Two international research collaborations were created that attempting to address how the pandemic affected vascular surgery patients, the COvid-19 Vascular sERvice (COVER) Study^4,5^ and the Vascular Surgery COVID-19 Collaborative (VASCC)^6^.

Acute limb ischaemia is one of the manifestations of COVID-19 infection. The European Society for Vascular Surgery^7^ published the first-ever clinical practice guidelines on acute limb ischaemia in February 2020, at the verge of the pandemic. A year and a half into the pandemic we performed a scoping review of the literature, and updated the guidelines in light of the pandemic^8^. We had hoped that the mentioned research collaborations would help us, but in fact they added nothing of importance to our review. Less than half of the vascular surgical units in the UK had reported anything to COVER, and VASCC had published nothing at all. The COVER publication^5^ on vascular services during the first wave of the pandemic did not even mention acute limb ischaemia.

In contrast, data from the Swedvasc registry comparing population-based data on vascular surgical procedures in 2020 with those of 2017–2019 were submitted in April 2021^9^. The Swedvasc registry has been validated on multiple occasions since its creation in 1987, showing excellent external and internal validity. Similarly, the UK National Vascular Registry published multiple short reports on how the pandemic affected vascular surgery in the UK (https://www.vsqip.org.uk/content/uploads/2020/11/NVR-Short-Report-Covid-19.pdf). It took many years to harmonize variables and definitions with other countries participating in the Vascunet collaboration, founded in 1997. It is naive to think that creating a database in which ‘the first protocol was developed in 3 days’, and to invite everybody to participate, without checking whether submission of data was neither complete nor correct, could be the basis of high-quality research. There are no short cuts to performing research that helps to improve clinical practice.

In summary, I agree with Li and Bhangu that we should avoid single-centre retrospective studies and spend energy creating quality data sets instead. We must be aware of the limitations of rapidly established large collaborative research initiatives, in order not to jump from one ineffective research model to another. Population-based and well validated data are more robust. Authorship rules should be respected; they are not elitist, but fair in giving both merit and responsibility to those who did the hard work. Countries such as the UK, Scandinavia, and the Netherlands have a great asset in our healthcare systems. They are assets not only for our patients, but also for the advancement of clinical science on a global level.

*Disclosure*. The author declares no conflict of interest
